# Mathematical models for devising the optimal Ebola virus disease eradication

**DOI:** 10.1186/s12967-017-1224-6

**Published:** 2017-06-01

**Authors:** Shuo Jiang, Kaiqin Wang, Chaoqun Li, Guangbin Hong, Xuan Zhang, Menglin Shan, Hongbin Li, Jin Wang

**Affiliations:** 1 Scientific Research Center, Shanghai Public Health Clinical Center, Fudan University, 2901 Caolang Road, Jinshan District, Shanghai, 201508 China; 2 Faculty of Business and Economics, The University of Hong Kong, Pokfulam, Hong Kong China; 3 Department of Dermatology, First Affiliated Hospital of Kunming Medical University, 295 Xichang Road, Kunming, 650032 Yunnan China; 4 Department of Infectious Diseases, Shanghai Public Health Clinical Center, Fudan University, Shanghai, China; 5 Department of Economics, Tufts University, 8 Upper Campus Road, Braker Hall, Medford, MA 02155 USA

**Keywords:** Ebola, Spread, Eradication pathways, Hospital isolation, Mathematical model

## Abstract

**Background:**

The 2014–2015 epidemic of Ebola virus disease (EVD) in West Africa defines an unprecedented health threat for human.

**Methods:**

We construct a mathematical model to devise the optimal Ebola virus disease eradication plan. We used mathematical model to investigate the numerical spread of Ebola and eradication pathways, further fit our model against the real total cases data and calculated infection rate as 1.754.

**Results:**

With incorporating hospital isolation and application of medication in our model and analyzing their effect on resisting the spread, we demonstrate the second peak of 10,029 total cases in 23 days, and expect to eradicate EVD in 285 days. Using the regional spread of EVD with our transmission model analysis, we analyzed the numbers of new infections through four important transmission paths including household, community, hospital and unsafe funeral.

**Conclusions:**

Based on the result of the model, we find out the key paths in different situations and propose our suggestion to control regional transmission. We fully considers Ebola characteristics, economic and time optimization, dynamic factors and local condition constraints, and to make our plan realistic, sensible and feasible.

**Electronic supplementary material:**

The online version of this article (doi:10.1186/s12967-017-1224-6) contains supplementary material, which is available to authorized users.

## Background

The current Ebola virus disease (EVD) outbreak in West Africa is now the largest yet documented and has caught worldwide attention [1, 2] with the countries of Guinea, Liberia, and Sierra Leone most affected [3]. The current Ebola virus (EBOV) outbreak in western Africa has caused more than 28,000 infections and over 11,100 deaths by Dec, 2015 [4] and the epidemic is increasing rapidly due to socioeconomic disadvantage and health system inadequacies in the three main affected countries [5, 6]. This is by far the largest outbreak of the virus in history, the virus spreads through human-to-human transmission [7, 8]. A study of the current EBOV outbreak showed that the case fatality rate was 34.7% overall and was higher for patients with stage 3 disease with neurological symptoms (66.7%) [9]. The surveillance and outbreak response management system architecture has been used to support the control of the EVD outbreak in West Africa as framework for Ebola virus disease outbreak modeling [10]. The new discoveries of Ebola medication announced by the World Medical Association (WMA) brings hope to the Ebola infected area. There are various modeling studies of the EVD epidemic have been reported using a wide range of quantitative approaches and obtaining analysis of the reproduction number of Ebola outbreak [11–16]. Household structured epidemic models have also provided some interesting insights of demographic determinants of Ebola epidemic risk [17]. These mathematical models were developed for the largest epidemics reported and involved in original EVD epidemiological data and genomic data [2, 13, 18–28], which predicted many more cases than actually occurred, some models produced more accurate predictions, and others yielded valuable insights. Recently, a system review and meta-analysis of 66 mathematical modeling studies of the EVD epidemic published in the peer-reviewed literature has been applied to assess these key models, data and model performance [29].

Further to improve forecast accuracy and investigate the spread and eradication pathways of EVD, a mathematical model to devise the optimal Ebola eradication plan for implementation by local governments, pharmaceutical companies, logistics companies, and international organizations needs to take into consideration medical condition improvement, education campaign, personal protection, regulation promulgation, and other contributing factors and considered Ebola characteristics and its spread trend, local condition constraints, and economic optimization to make the models realistic, sensible and feasible. The purpose of our study reported here was to develop a mathematical model to devise the optimal Ebola virus disease eradication plan. Here, we only choose Liberia as our study country in our model, which is flexible for other countries in the same way and not only limited to Liberia.

## Methods

### Characteristics of Ebola

For feasibility and usefulness of our approach, the important attributes of the Ebola virus and EVD before constructing our models were be summarized and included (1) Origin: the bat species, fruit bats in particular, are considered to be the natural reservoir of Ebola virus. Severe forest loss in Africa in recent years has brought potentially infectious wild animals into closer contact with human settlements; this increases the risk of virus transmission from wild animals to people; (2) transmission: Ebola spreads mainly through body fluids. In each outbreak, the virus is first introduced into the human population through close contact with bodily fluids of infected wild animals or infected people [30]. The common symptoms of EVD include diarrhea, fatigue, fever, muscle pain, severe headache, abdominal or stomach pain, vomiting, and unexplained hemorrhage. The course of EVD shows three phases in each case: (1) Incubation phase: infected patients generally don’t show any particular symptoms of EVD and patients don’t transmit the virus in this period; (2) Early infective phase: infected patients begin showing explicit symptoms such as fever and fatigue, and the EVD medication developed by WMA can cure the disease; (3) Advanced infective phase: infected patients are close to death, and cannot be cured by existing EVD medication.

### Assumptions of modeling for EVD analysis

For the model for prediction of the numerical spread of Ebola and regional transmission of Ebola, given that births and deaths affect the total population and the duration of the outbreak is short, we won’t take the change of total population in consideration. Firstly, we assume the population of an infected region remains constant in the ongoing outbreak, which enables us to focus on the analysis of infected people. Since people in the incubation phase and early infective phase can be cured by EVD medication, and the incubation phase is assumed impossible to detect. Next, we assume that the vaccine could not provide 100% protection from the Ebola virus, which its true effective vaccination rate was at between 75 and 100% [31]. At the same time, it is expected that the treatment time of EVD will gradually reduce with improving medical condition in these countries.

### Model for prediction of the numerical spread of Ebola

**Fig. 1:**
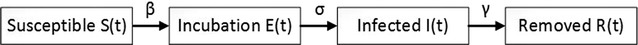
Flow chart of the simplified model

A series of differential equations systems was built to study the trend of Ebola total cases and construct a basic model based on traditional SEIR model by fitting our model against the actual total cases data. However, the effect of hospital isolation and application of medication are usually neglected. So we build a modified epidemic model which takes hospital isolation, Ebola drug and vaccine into account, and later verified the result with Monte Carlo Algorithm. We are concerned about the numerical spread pattern of Ebola and the function of these resistance methods against the spread of the disease. We assume that (1) the population of an infected region remains constant in the ongoing outbreak; (2) vaccine recipients and recovered patients develop complete immunity to Ebola virus; and (3) 98 of every 100 people who are vaccinated will successfully develop immunity against the disease. In this study, we divide the total population into seven groups in our model including (1) Susceptible group (*S*), no immunity against the disease and are very likely to be infected in direct contact with the infective people. *S* denotes the number of people in the susceptible group; (2) Incubation group (*E*), infected and cured by taking Ebola drugs, but had not displayed any explicit symptom. *E* denotes the number of people in the incubation group; (3) Early stage infected group (*I*_*E*_), displayed explicit symptoms of EVD and could transmit the virus to susceptible people and be cured by the EVD drug. We assume this phase lasts 3 days; (4) Advanced infected group (*I*_*L*_), displayed explicit symptoms of EVD and could transmit the virus to susceptible people, but cannot be cured by the EVD drug. We assume this phase lasts 2.61 days; (5) Removed group (*R*), died of the disease or survived the disease; (6) Hospital isolation group (*H*), shifted from the infective group and isolated from the susceptible people, and Ebola drugs are only for patients in hospital isolation; (7) Immunity group (*M*), gain complete immunity against Ebola virus and could either get the effective immunity through vaccine injection or recovery from the disease. Other related parameters are shown in the Table 1. This part shows how we obtain the value of the critical parameter: the infection rate β. The model introduced below can be displayed in the flow chart shown in Fig. 1. Note that this model is simplified compared to our “numerical spread” model, due to the availability of data and the solvability of the differential equation system model. The simplified model is listed below:

$$\frac{dS}{dt} = - \beta S\frac{I}{N}$$

$$\frac{dE}{dt} = \beta S\frac{I}{N} - \sigma E$$

$$\frac{dI}{dt} = \sigma E - \gamma I$$

$$\frac{dR}{dt} = \gamma I$$

**Table 1:** Definition of parameters for the modified epidemic model

Parameters	Definition
*á*	The isolation rate, i.e. the rate of people moved from the infective group to the hospital isolated group
$\hat{a}$	The infection rate, i.e. the rate of the susceptible population get infected
$\tilde{a}_{E}$	The outflow rate of early stage infected group, i.e. the rate that early infected people turn advanced infected
$\tilde{a}_{L}$	The outflow rate of advanced infected group, i.e. the rate that advanced infected people die of the disease
*ό*	The outflow rate of the incubation group to the early stage infective group
*ù*	The outflow rate of the isolation group, that is the rate of isolated patients get cured.
*N*	The total population
Nc(t)	Number of other household in other countries of one un-hospitalized infected people at time t;
*c*	Comprehensive treatment and immunization rate
$\Delta$	The ratio of the case input (<0) or the output (>0)

**Fig. 2:**
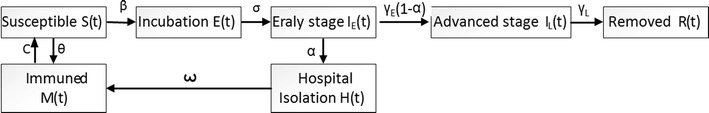
Flow chart of the modified epidemic model

The differential equation system model is numerically solvable given the values of the parameters (γ, σ and β, and note that the former two parameters are known). We apply the grid search algorithm to determine the value of β. The objective function for the algorithm is

$${\min}{_{\beta}} \sum\limits_{i = 1}^{n} {\left( {f_{i} - d_{i} } \right)^{2} }$$

where *f*_*i*_ denotes the numerical solution of the model at date i as the total cases, and *d*_*i*_ denotes the true value of it. The population flow between these groups is shown in Fig. 2, and we present the differential equations system of our model as follows:

1 $$\frac{dS}{dt} = - \beta S\frac{{I_{E} + I_{L} }}{N} - \theta S + cM$$

2 $$\frac{dE}{dt} = \beta S\frac{{I_{E} + I_{L} }}{N} - \sigma E$$

3 $$\frac{{dI_{E} }}{dt} = \sigma E - \gamma_{E} (1 - \alpha )I_{E} - \alpha I_{E}$$

4 $$\frac{{dI_{L} }}{dt} = \gamma_{E} (1 - \alpha )I_{E} - \gamma_{L} I_{L}$$

5 $$\frac{dH}{dt} = \alpha I_{E} - \omega H$$

6 $$\frac{dM}{dt} = \theta S + \omega H - cM$$

7 $$\frac{dR}{dt} = \gamma_{L} I_{L}$$

### Model for regional transmission of Ebola

**Fig. 3:**
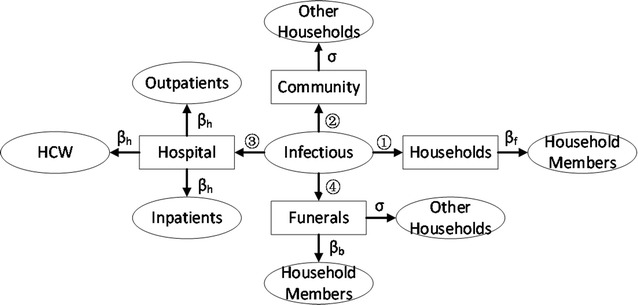
Illustration of regional Ebola transmission paths

There are four important transmission paths, which are through household, community, hospital and unsafe funeral for calculating daily infection cases through each path. We find out the key paths in different situations and propose our suggestion to control regional transmission, and use a transmission model to study the regional spread of Ebola virus [18]. Here, we assume that people in isolation group cannot transmit Ebola virus to others, but it’s not true in reality. We break down the regional spread consider four Ebola transmission paths more practically shown as follows. (1) Transmission within households: from un-hospitalized infective people to his or her household; (2) Transmission in the general community: from unhospitalized infected people to other household in his or her community. (3) Transmission in hospitals: from hospitalized infected people to health care workers (HCWs), From hospitalized infected people to other not-Ebola-infected patients in hospitals and from hospitalized infective people to his or her uninfected household in the hospital who take care of them; (4) Transmission in unsafe funeral: To household of the dead people and other household. We illustrate the important transmission paths with Fig. 3. The letters on the arrow are the transmission rate from the infected people to the health people. And we denote the number of household of one un-hospitalized infected people, other household in the community of one un-hospitalized infected people and contacts with hospitalized infected people at time *t as N*_*f(t)*_ *, N*_*a(t)*_ and *N*_*h(t)*_, shown in the Table 2.

**Table 2:** Definition of parameters for model two

Parameter	Definition
ë(t)	Infection force at time t
â_f_	Infection rate of un-hospitalized infected people with the household
*ό*	Ratio of the transmission rate in the other households in the same community to that within the household
${\hat{\text{a}}}_{\text{h}}$	Infection rate of hospitalized infected people
${\hat{\text{a}}}_{\text{b}}$	Infection rate of an unsafe funeral
N_f(t)_	Number of household of one un-hospitalized infected people at time t
N_a(t)_	Number of other household in the community of one un-hospitalized infected people at time t
N_h(t)_	Number of contacts with hospitalized infected people at time t
I_(t)_	Number of infected people at time t
∝	Isolation rate
f	Fatality rate
u	Unsafe funeral ratio

## Results

### Estimation of the numerical spread of Ebola

An SEIR (susceptible-exposed-infectious-recovered) model was previously established for the Ebola endemic, which provided the estimates of reproduction numbers in Guinea, Sierra Leone and Liberia (Additional file 1: Table S1) [30]. However, it didn’t take control interventions into consideration. We take the method as estimate infection rate ${\hat{\text{a}}}$ in model; then consider the function of resistance methods in our modified model, which will offer more reference for government planning of Ebola eradication. Since the period that people will stay in the incubation phase and in the infective phase differed little, we take the incubation period and infective period (*1/ό* = 5.3 days and $1/\tilde{a} = 5.61$) days in previous estimates from an outbreak of EVD in Congo in 1995 with our model [32]. We assume the value of ${\hat{\text{a}}}$ remains constant in the studied period. In this study, we first fitted our model against the real EVD total cases data of Liberia from 2nd July 2014–28th August 2014. We obtain the value of infection rate ${\hat{\text{a}}}$ as 1.754 in our basic SEIR model and Monte Carlo algorithm given the value of other parameters in the differential equations system, which our estimating value of infection rate ${\hat{\text{a}}}$ is a little higher, but acceptable, comparing with other results of related research. For examples, Althaus CL estimated the value to be 1.59 and WHO Ebola Response Team estimated the value to be 1.51 (both for Liberia) [33].

### The effect of vaccination on eradicating Ebola

**Fig. 4:**
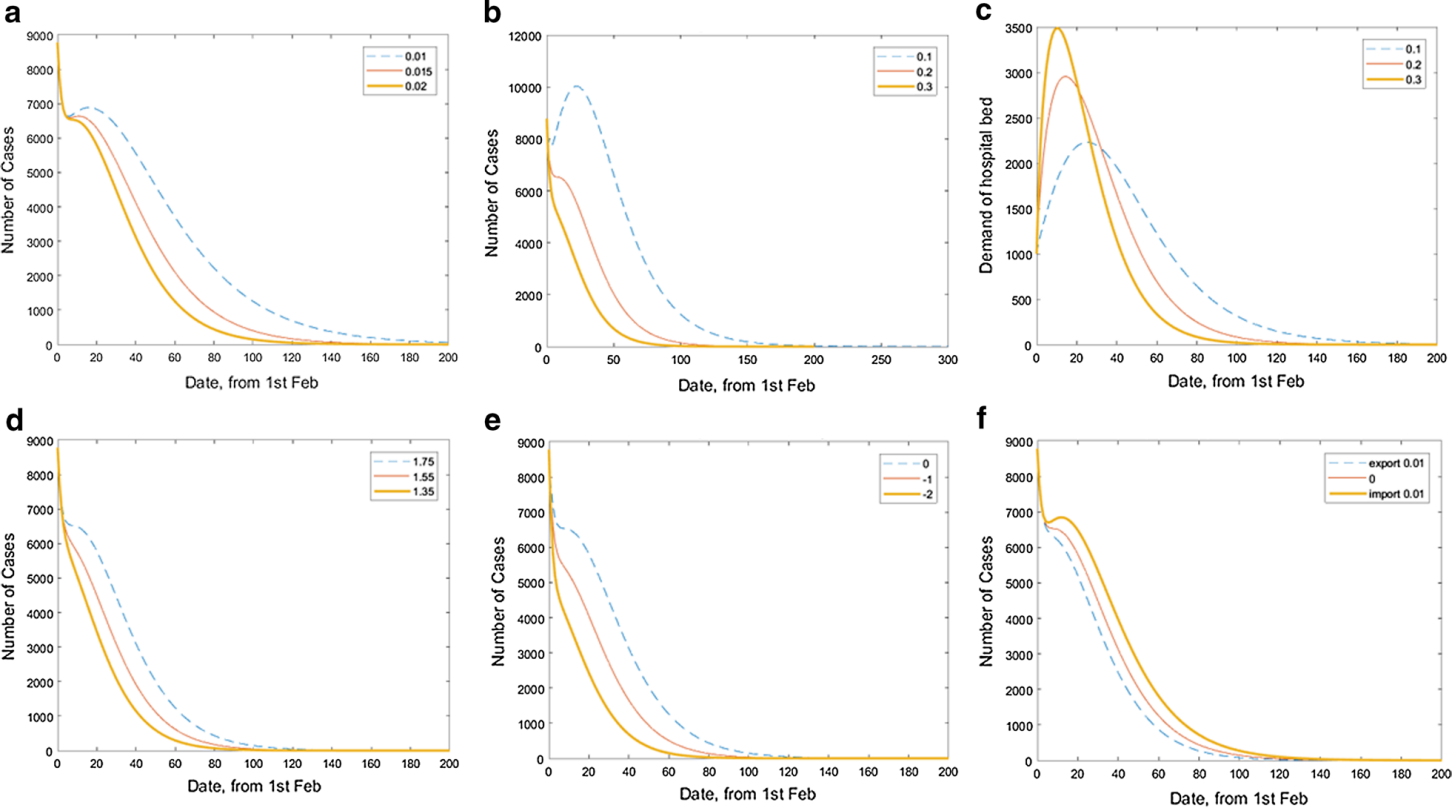
Impact of **a** vaccination rate, **b** isolation rate on eradicating Ebola and **c** isolation rate on hospital beds demand, and **d** impact of infection rate on eradicating Ebola, and **e** impact of treatment time on eradicating Ebola, and **f** impact of imported or exported cases rate on eradicating Ebola

Next, we predict the eradication process of Ebola. We set 1st Feb 2015 as t = 0, and use the Ebola disease statistics of t = 0 on WHO as the initial state of our model. As is stated before, we focus on the function of resistance methods, namely hospital isolation, Ebola medication and medical protection against the spread of the disease. Thus, we change the value of relevant parameters (vaccination rate, isolation rate and infection rate) to stimulate different eradication process under these settings. For analysis of vaccination rate, we set á = 0.2 and vary the vaccination rate è. The results in Fig. 4a show that the bigger the vaccination rate è is, the sooner the disease can be eradicated quite apparently and sensibly. Based on our prediction, which set è = 0.02 and á = 0.2, we can expect to eradicate the disease in 188 days (number of total cases <1). The actual eradication process should be shorter because when there are a small number of infected people, we can put all of them in hospital isolation and cure the with Ebola drugs. That is á = 1 when total cases number is small enough. This should also apply to our analysis of á and ${\hat{\text{a}}}$ later.

### The effect of hospital isolation on eradicating Ebola

For analysis of isolation rate, we tested the effect of hospital isolation on eradicating Ebola. We set è = 0.01 and vary the isolation rate á. The general trend is very similar to our analysis about the vaccination rate in Fig. 4b, which the bigger the isolation rate á is, the sooner the disease can be eradicated; and the eradication process can be divided into three phases. When we set è = 0.02 and á = 0.1, the number of total cases decreases to 7706 in 4 days, increases to the second peak of 10,029 total cases in 23 days; we can expect to eradicate the disease in 285 days (number of total cases <1). The actual eradication process should be shorter as discussed above. Moreover, high isolation rate can resist the second rise (like á = 0.3). The determination of isolation rate á is subject to the medical condition of one country, i.e. the total number of hospital beds of Ebola. The demand for hospital beds by time is shown in the Fig. 4c. We assume there are 1000 isolated people in hospital on 1st Feb. We can notice from the figure above that high isolation rate means a faster rise and more quantity in demand of hospital beds. The numbers of maximum hospital beds needed are 3488 in 11 days, 2954 in 15 days and 2230 in 26 days for á = 0.3, á = 0.2, and á = 0.1, respectively. Therefore, each country can determine their isolation rate á based on their medical condition, i.e. the number of hospital beds.

### Effect of Ebola infection rate on eradicating Ebola

Further, we set the value of the infection rate â as 1.754 in accordance with our model result. In fact, the infection rate can be controlled by public medical education and protection. We analysis the trend of total cases number with different ${\hat{\text{a}}}$ value (${\hat{\text{a}}} = 1.75,\,1.55,\,1.35$), we set á = 0.2 and è = 0.02. The result is shown in Fig. 4d. The general trend is also similar: (1) The smaller the infection rate ${\hat{\text{a}}}$, the sooner the disease can be eradicated: (2) The eradication process also can be divided into three phases as we have discussed above and low infection rate can resist the second rise.

### Effect of Ebola treatment time on eradicating Ebola

We think that the average time from symptom onset to medical treatment will be shortened with the improvement of treatment, which will affect eradicating Ebola. Firstly, we simulate the average time from symptom onset to medical treatment by shortening the treatment time. Since treatment of EVD involves both of early and late stages in infection of EBOV, we set á = 0.2 and è = 0.02, and assume that treatment time will be shortened both in the early and late stages in infection of EBOV. Here, we simulate three models: one is the original model of the early treatment time (γE = 3 days) and late treatment time (γL = 2.61 days); if all the assuming is same, there are other two simulation results (Fig. 4e), which the early and late treatment time in the two models is reduced by 1/2 or one day. We investigate that the time of eradicating Ebola is shortened if the treatment time is shortened, and expect to eradicate EVD in 188, 152 and 120 days in the three models if the case is less than 1, respectively.

### Effect of Ebola imported or exported cases rate on eradicating Ebola

Imported or exported cases of Ebola from one country to another country will affect the eradicating Ebola of the two countries [34]. To simulate this situation, the second equation in the above model is changed and listed below 8.

8 $$\frac{dE}{dt} = \beta S\frac{{I_{E} + I_{L} }}{N} - (\sigma + \vartriangle )E$$

During the outbreak of Ebola in Liberian in 2014, it is estimated that the imported and exported cases does not exceed 1% [34]. $\Delta$ represents the ratio of the imported case (n < 0) or the exported case (n > 0). Here, we set á = 0.2 and è = 0.02 and simulate the three situations: one is no imported and exported case; one is only exported cases (1%) or imported cases (1%), which were shown in Fig. 4f. For eradicating Ebola, we can notice that the imported and exported effects are presented in two aspects: (1) the exported weakens the second peak, as the imported will strengthen the second peak; (2) the imported extends the outbreak time of EVD, and the exported can reduce its outbreak time. However, there is little difference among these three situations.

### Force of infection of each Ebola transmission path

For each Ebola transmission path, at time t, we can calculate the force of infection as below:

9 $${{\uplambda }}_{1} \left( t \right) = \frac{{\beta_{f} }}{{N_{f} \left( t \right)}}$$

10 $${{\uplambda }}_{2} \left( t \right) = \frac{{\sigma \beta_{f} }}{{N_{a} \left( t \right)}}$$

11 $${{\uplambda }}_{3} \left( t \right) = \frac{{\beta_{h} }}{{N_{h} \left( t \right)}}$$

12 $${{\uplambda }}_{4} \left( t \right) = \frac{{\beta_{b} }}{{N_{f} \left( t \right)}}$$

13 $${{\uplambda }}_{5} \left( t \right) = \frac{{\sigma \beta_{b} }}{{N_{a} \left( t \right)}}$$

For calculating the infection force of each Ebola transmission paths, we can get the infection cases, which are *(1* *−* *á)I(t)* $\hat{a}_{f}$ */N*_*f(t)*_ *, (1* − *á)I(t) ó* $\hat{a}_{f}$ */N*_*a(t)*_ *, áI(t)* $\hat{a}_{h}$ */N*_*h(t)*_ and *ufI(t)(* $\hat{a}_{b}$ */N*_*f(t)*_ + *ó* $\hat{a}_{b}$ */N*_*a(t)*_ *)* in the four transmission paths and the comprehensive information presented in Table 3.

**Table 3:** The analysis of infected people each day by different transmission paths

Paths	Household	Community	Hospital	Funeral
Infected people	*(1* − *á)I(t)*	*(1* − *á)I(t)*	*áI(t)*	*ufI(t)*
Infection force ë(t)	*â*_*f*_ */N*_*f(t)*_	*ó* $\hat{a}_{f}$ */N*_*a(t)*_	$\hat{a}_{h}$ */N*_*h(t)*_	$\hat{a}_{b}$ */N*_*f(t)*_ + *ó* $\hat{a}_{b}$ */N*_*a(t)*_
Total infection cases	*(1* − *á)I(t)* $\hat{a}_{f}$ */N*_*f(t)*_	*(1* − *á)I(t) ó* $\hat{a}_{f}$ */N*_*a(t)*_	*áI(t)* $\hat{a}_{h}$ */N*_*h(t)*_	$ufI(t)\left( {\hat{a} _{b} /N_{{f(t)}} + \acute{o} \hat{a} _{b} /N_{{a(t)}} } \right)$

We take $\hat{a}_{f}$= *0.12, ó* = *0.79,* $\hat{a}_{h}$ = *0.44,* $\hat{a}_{b}$ = *0.12,* and *f* = *0.7* [35]. And we assume an un-hospitalized infected people has an average of 5 households and an average of 5 other households, and they contact an average of 10 people at each time, thus *N*_*f(t)*_ = *N*_*a(t)*_ = 5, and *N*_*h(t)*_ = 10 *I(t*_*0*_ *)* = *2000* in one supposed region. Through our simulation, the number of infection cases was obtained though different paths in regional spread of Ebola by setting µ = 0.3 and vary isolation rate á with 0.3, 0.5 and 0.7, which shown in Table 4. When isolation rate is small, household transmission path is the most important way of Ebola spread. When isolation rate is big, hospital transmission path becomes the most important way. This means the most important path varies in different situations.

**Table 4:** Number of new infection cases through different paths with different values for hospital isolation rate (á)

Paths\isolation rate	á = 0.3	á = 0.5	á = 0.7
Household	33.6	24.0	14.4
Community	26.5	19.0	11.4
Hospital	13.2	22.0	30.8
Unsafe funeral	18.0	17.0	16.3
Total cases	91.3	82.0	72.9

## Discussion

People in Western Africa are living in an abyss of misery because of the ongoing Ebola outbreak. Scientist have joyfully announced the invention of Ebola medication, including Ebola drug and vaccine and call on joint effort from all parties involved to eradicate Ebola as soon as possible [36, 37]. Local governments have the responsibility to set the deadline for the final eradication, and the Ebola epidemic in Western Africa rather reveals fundamental failures in establishing health policies in Western Africa [38]. Considering regional spread of Ebola is very important, prevention of infection on important paths can decrease the infection rate of Ebola. Thus, effective Ebola eradication must involve active cooperation between the government, pharmaceutical companies and international organizations. In this study, our approach can make eradication plans with different local constraints and eradication time goals. It shows great adaptability and feasibility and takes the efficient Ebola eradication as our first priority, and give weight on economic optimization.

Firstly, we consider both numerical and regional spread of Ebola virus, and our approach is based on Ebola transmission mechanism and characteristics. We assume comprehensive treatment and immunization rate is 98%. We further analyzed the effect of various á, è, ${\hat{\text{a}}}$, $\gamma_{E}$,$\gamma_{L}$ and $\Delta$ values on Ebola eradication in this study, which provides a good instruction on the eradication effort for the local government. First, it must maintain its vigilance in the early phases and enhance detection to predict the potential second epidemic peak. Large quantities of drugs and hospital beds should be prepared in advance to handle the second peak. Second, high á, è, $\Delta$value and low ${\hat{\text{a}}}$, $\gamma_{E}$, $\gamma_{L}$ value can resist the second rise of total cases number. But their values are dependent on medical, educational, economic, transportation and cultural factors of each country, which should be balanced by local government for contributing to a faster eradication plan. Third, local government should keep tight monitor on the epidemic situation and must take immediate measure against it, such as delivery of drugs and vaccines in large quantity, and immediate isolation of newly infected people, in case there may be unexpected outbreaks in the eradication process. Regional transmission is the root of Ebola spread. We find out that the four important transmission paths are through household, community, hospital and unsafe funeral. The governments should look into these paths and control transmission through each. This can be managed by widespread Ebola protection education campaign, professional funeral treatment, protection regulation.

**Fig. 5:**
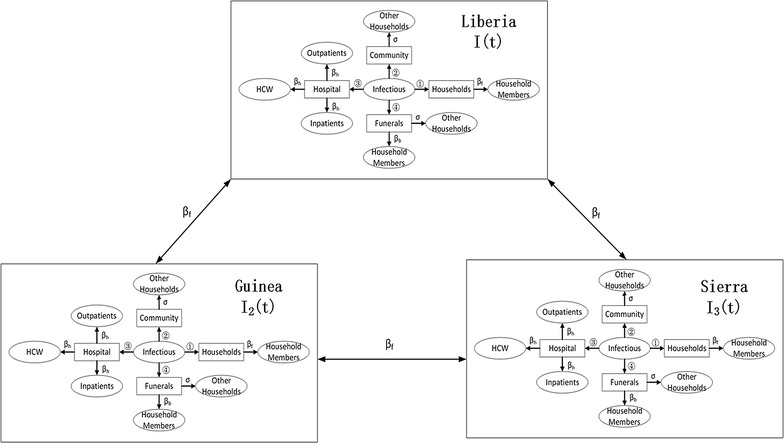
The eradicating Ebola model of the three countries (Liberia, Guinea and Sierra)

**Fig. 6:**
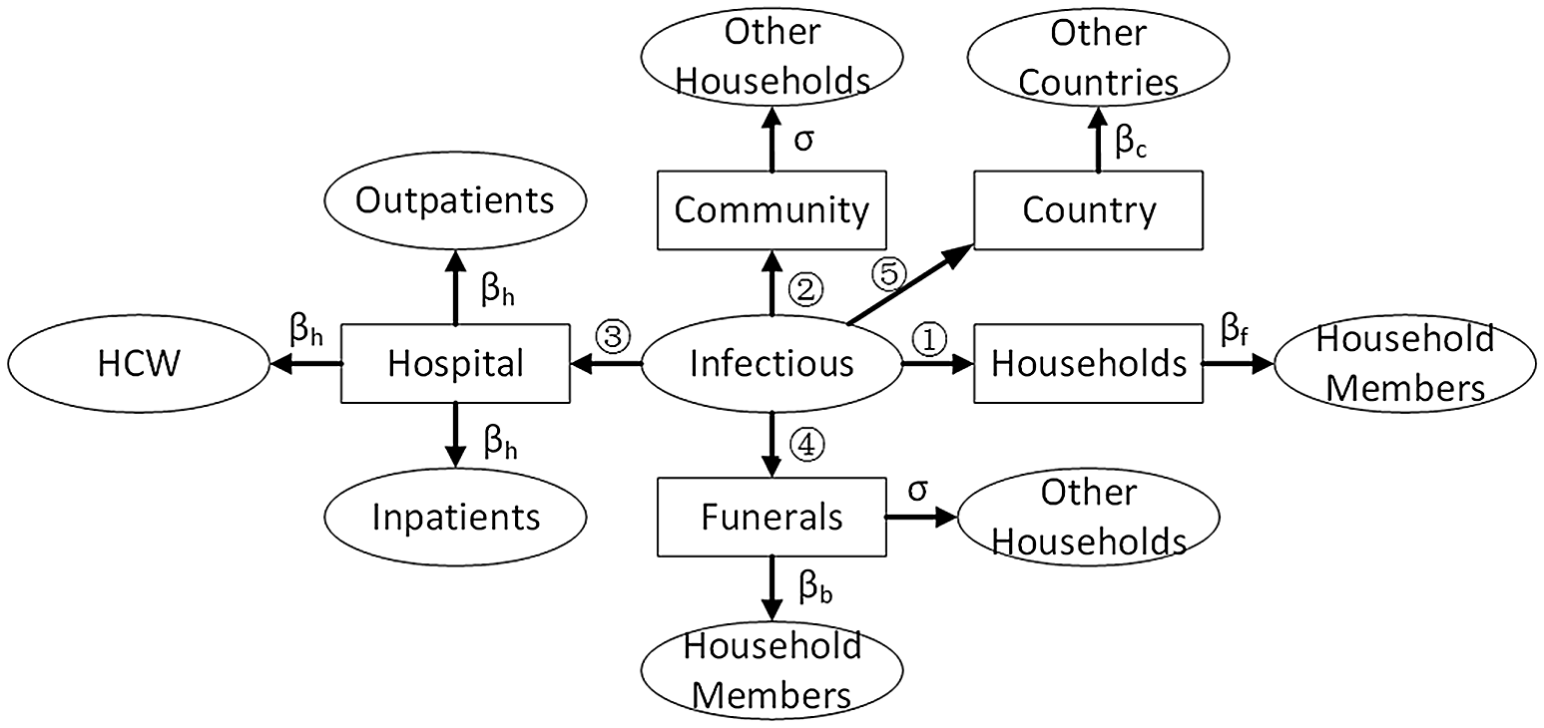
Illustration of regional Ebola transmission paths in Liberia (the community in the transmission path)

Further, we analyze the changes in their prediction/eradication model when they combine the three countries into a single region. We demonstrated that prediction model is unchanged. The three countries should be predicted separately and then added the sum. Because the situation of Ebola virus infection in each country is different, and infection between countries is less likely to be exposed. Next, for eradication model, the three countries are considered together, the model is presented in Fig. 5. We assume that the total infection of Guinea and Sierra is I2 (t) and I3 (t). For example, the community in the transmission path in Liberia will be divided into other households in the same country and other households in the different countries, as shown in Fig. 6. Finally, we can get infected people = I2(t) + I3(t); Infection force λ(t) = βf/Nc(t); Total infected cases = [I2(t) + I3(t)] * βf/Nc(t), which Nc(t) is the number of other household in other countries of one un-hospitalized infected people at time t.

Based on the model, to lower the infection force of the four paths, such as house hold, community, hospital and unsafe funeral, house hold pathway needs a widespread education campaign on the basic knowledge of Ebola throughout the region because early detection of Ebola symptoms and isolation can better prevent other household from being infected. Community pathway suggested that people should meet as fewer people as possible. When meeting with other people, they should take protective actions, like wearing a respirator. Hospital pathway should be enough isolation of Ebola patients from other patients to decrease infection, more concerned about protecting doctors and nurses from being infected, especially when incubation rate is higher, and should limit or even forbidden household company in the hospital [39, 40].

## Conclusions

In summary, we have found out the key paths in different situations and proposed our suggestion to control regional transmission. Ebola eradication needs systematic thinking, effective hospital isolation, and effective EVD drug and vaccination. The desired eradication deadline based on our models can determine the demand of the three weapons against Ebola virus.

## Additional file

**Additional file 1: Table S1.** Ebola cumulative reported cases.
