# Supplementary material for: Mathematical models for devising the optimal Ebola virus disease eradication
Source: J Transl Med. 2017 Jun 1;15:124. doi: 10.1186/s12967-017-1224-6 (PMC5452395; doi:10.1186/s12967-017-1224-6)
Supplement: Supplementary file 1 — Additional file 1: Table S1. Ebola cumulative reported cases. [file 12967_2017_1224_MOESM1_ESM.pdf]

Mathematical Models for Devising the Optimal Ebola Virus Disease Eradication

Shuo Jiang[1, 2,#], Kaiqin Wang[3,#], Chaoqun, Li[4, #], Guangbin Hong[5,#], Xuan Zhang[1], MenglinShan[1], Hongbin Li[3], Jin Wang[1,*]

[1]Scientific Research Center, Shanghai Public Health Clinical Center, Fudan University, 2901 Caolang Road, Jinshan District, Shanghai 201508, China; [2]Faculty of Business and Economics, The University of Hong Kong, Pokfulam, Hong Kong, China; [3]Department of Dermatology, First Affiliated Hospital of Kunming Medical University, 295 Xichang Road, Kunming, 650032, Yunnan, China; [4]Department of Infectious Diseases, Shanghai Public Health Clinical Center, Fudan University, Shanghai, China; [5]Department of Economics, Tufts University, 8 Upper Campus Road, Braker Hall, Medford, MA 02155, USA.

Supplementary Tables:

1. Table S1：Ebola cumulative reported cases in Guinea, Liberia, and Sierra Leone.

WHO report date	Total Cases, Guinea	Total Deaths, Guinea	Total Cases, Liberia	Total Deaths, Liberia	Total Cases, Sierra Leone	Total Deaths, Sierra Leone
2014/3/1	0	0	0	0	0	0
2014/3/2	0	0	0	0	0	0
2014/3/3	0	0	0	0	0	0
2014/3/4	0	0	0	0	0	0
2014/3/5	0	0	0	0	0	0
2014/3/6	0	0	0	0	0	0
2014/3/7	0	0	0	0	0	0
2014/3/8	0	0	0	0	0	0
2014/3/9	0	0	0	0	0	0

2014/3/10	0	0	0	0	0	0
2014/3/11	0	0	0	0	0	0
2014/3/12	0	0	0	0	0	0
2014/3/13	0	0	0	0	0	0
2014/3/14	0	0	0	0	0	0
2014/3/15	0	0	0	0	0	0
2014/3/16	0	0	0	0	0	0
2014/3/17	0	0	0	0	0	0
2014/3/18	0	0	0	0	0	0
2014/3/19	0	0	0	0	0	0
2014/3/20	0	0	0	0	0	0
2014/3/21	0	0	0	0	0	0
2014/3/22	0	0	0	0	0	0
2014/3/23	0	0	0	0	0	0
2014/3/24	0	0	0	0	0	0
2014/3/25	86	59	0	0	0	0
2014/3/26	86	60	0	0	0	0
2014/3/27	103	66	8	6	6	5
2014/3/31	112	70	8	6	0	0
2014/4/1	122	80	8	2	0	0
2014/4/2	127	83	8	5	0	0
2014/4/7	151	95	18	7	0	0
2014/4/10	157	101	22	14	0	0
2014/4/17	197	122	27	13	0	0
2014/4/21	203	129	27	13	0	0
2014/4/23	208	136	34	11	0	0
2014/4/30	221	146	13	11	0	0
2014/5/5	231	155	13	11	0	0
2014/5/14	233	157	12	11	0	0
2014/5/23	258	174	12	9	0	0
2014/5/27	258	174	12	9	1	4
2014/5/28	281	186	12	9	16	5
2014/6/2	291	193	13	9	50	6
2014/6/5	344	215	13	9	81	7
2014/6/10	372	236	15	10	89	7
2014/6/11	376	241	15	10	117	19
2014/6/18	398	264	33	24	97	49
2014/6/24	390	270	51	34	158	34
2014/7/2	413	303	107	65	239	99
2014/7/7	412	305	115	75	252	101
2014/7/8	408	307	131	84	305	127
2014/7/14	409	309	142	88	337	142
2014/7/16	406	304	172	105	386	192

2014/7/21	410	310	196	116	442	206
2014/7/24	415	314	224	127	454	219
2014/7/28	427	319	249	129	525	224
2014/7/31	460	339	329	156	533	233
2014/8/3	472	346	391	227	574	252
2014/8/4	485	358	486	255	646	273
2014/8/8	495	367	554	294	717	298
2014/8/12	506	373	599	323	730	315
2014/8/13	510	377	670	355	783	334
2014/8/15	519	380	786	348	810	348
2014/8/19	543	394	834	466	848	365
2014/8/21	579	396	972	576	907	374
2014/8/22	607	406	1082	624	910	392
2014/8/28	648	430	1378	694	1026	422
2014/9/6	812	517	1871	1089	1261	491
2014/9/8	862	555	2046	1224	1361	509
2014/9/12	861	557	2081	1137	1424	524
2014/9/16	936	595	2407	1296	1620	562
2014/9/18	942	601	2710	1459	1673	562
2014/9/22	1008	632	3022	1578	1813	593
2014/9/24	1022	635	3280	1677	1940	597
2014/9/26	1074	648	3458	1830	2021	605
2014/10/1	1157	710	3696	1998	2304	622
2014/10/3	1199	739	3834	2069	2437	623
2014/10/8	1298	768	3924	2210	2789	879
2014/10/10	1350	778	4076	2316	2950	930
2014/10/15	1472	843	4249	2458	3252	1183
2014/10/17	1519	862	4262	2484	3410	1200
2014/10/22	1540	904	4665	2705	3706	1359
2014/10/25	1553	926	4665	2705	3896	1281
2014/10/29	1906	997	6535	2413	5235	1500
2014/10/31	1667	1018	6535	2413	5338	1510
2014/11/5	1731	1041	6525	2697	4759	1070
2014/11/7	1760	1054	6619	2766	4862	1130
2014/11/12	1878	1142	6822	2836	5368	1169

2014/11/14	1919	1166	6878	2812	5586	1187
2014/11/19	1971	1192	7069	2964	6073	1250
2014/11/21	2047	1214	7082	2963	6190	1267
2014/11/26	2134	1260	7168	3016	6599	1398
2014/11/28	2155	1312	7635	3145	7109	1530
2014/12/3	2164	1327	7635	3145	7312	1583
2014/12/10	2292	1428	7719	3177	7897	1768
2014/12/17	2416	1525	7797	3290	8356	2085
2014/12/24	2597	1607	7862	3384	9004	2582
2014/12/31	2707	1708	8018	3423	9446	2758
2015/1/7	2775	1781	8157	3496	9780	2943
2015/1/14	2806	1814	8331	3538	10124	3062
2015/1/21	2871	1876	8478	3605	10340	3145
2015/1/28	2917	1910	8622	3686	10518	3199
2015/2/4	2975	1944	8745	3746	10740	3276
